# Extensive glycoform heterogeneity in the gp120 envelope proteins used in the RV144 trial

**DOI:** 10.1186/1742-4690-9-S2-P296

**Published:** 2012-09-13

**Authors:** B Yu, JF Morales, SM O’Rourke, GP Tatsuno, PW Berman

**Affiliations:** 1 University of California, Santa Cruz, Santa Cruz, CA, USA

## Background

The AIDSVAX B/E vaccine used in the RV144 trial consisted of recombinant gp120s derived from the MN and A244 strains of HIV-1, both produced in CHO cells. In order to understand the correlates of protection and to design RV144 follow-up studies, it is important to characterize the vaccine immunogens and to know the extent to which newly manufactured gp120 subunit vaccines replicate the glycosylation of the AIDSVAX B/E vaccine immunogens. It has long been known that glycosylation affects antigenicity and immunogenicity. Recent data suggest that several epitopes recognized by broadly neutralizing antibodies are critically dependent on glycosylation in the gp120 V2 domain. In these studies we investigate the heterogeneity in net charge attributed to glycoform variation.

## Methods

Isoelectric focusing was used to analyze recombinant MN and A244 rgp120 proteins from the unformulated bulk used to manufacture the AIDSVAX B/E vaccine. We compared these proteins with MN and A244 rgp120 proteins freshly produced in 293 cells. Differences in the binding of monoclonal antibodies and soluble CD4 were measured by ELISA.

## Results

MN and A244 rgp120 proteins produced in CHO cells and 293 cells exhibit extensive heterogeneity in net charge due to glycosylation. More than 16 species of MN-rgp120 and 24 species of A244-rgp120 were identified. Proteins produced in CHO cells were distinctly more acidic than proteins produced in 293 cells. These differences affected the binding of ligands that targeted the CD4 binding site but not other regions of gp120.

## Conclusion

The rgp120 proteins used in the RV144 trial exhibited remarkable variation in net charge attributable to differences in glycosylation. The extent of gp120 glycosylation is cell-line dependent. Differences in glycosylation affect antibody binding and may represent a significant variable in the development of new antigens for RV144 follow-up studies and in neutralization assays that depend on pseudoviruses produced in 293 cells.

